# First report and molecular characterization of *Cryptosporidium* spp. in humans and animals in Khartoum state, Sudan

**DOI:** 10.14202/vetworld.2019.183-189

**Published:** 2019-01-31

**Authors:** Kaltoum Yagoub Adam, A. A. Ismail, M. A. Masri, A. A. Gameel

**Affiliations:** 1 Director General^’^s Office, Ministry of Animal Resources, Fisheries and Range Lands, Nyala, South Darfur State, Sudan; 2 Department of Pathology, Microbiology and Parasitology, College of Veterinary Medicine, Sudan University of Science and Technology, Khartoum, Sudan; 3 Department of Zoology, Faculty of Science, University of Khartoum, Khartoum, Sudan; 4 Department of Pathology, Faculty of Veterinary Medicine, University of Khartoum, Khartoum, Sudan

**Keywords:** *Cryptosporidium parvum*, nested polymerase chain reaction, staining techniques, Sudan, zoonotic

## Abstract

**Background and Aim:**

*Cryptosporidium* is recognized to infect several mammalian species as well as humans, causing substantial economic losses and serious public health concern. Infected animals can be a source of environmental contamination and human infections. In general, the occurrence of *Cryptosporidium* species in animals and human in Sudan and zoonotic importance is not well documented. This study aimed to identify *Cryptosporidium* spp. infecting different animal species and humans and to compare between different isolates obtained.

**Materials and Methods:**

To provide molecular information about *Cryptosporidium* in animals and humans, both modified Ziehl–Neelsen (MZN) specific stain and molecular assay were used. Concentration techniques followed by three protocols of DNA extraction were carried out. After microscopic screening of 263 fecal samples (goats [n=197], cattle [n=12], sheep [n=12], and human [n=42]), 61 positive and 30 negative, randomly selected samples were used in nested polymerase chain reaction (PCR) targeting part of the 18S RNA.

**Results:**

Nested PCR amplification confirmed 91.8% (56/61) of microscopic-positive samples. 8.2% (5/61) of negative samples by PCR (positive by microscopy) were considered false negatives. Sequencing followed by alignment of the 14 isolates indicated that all samples were identical (100%) and belonged to *Cryptosporidium parvum*.

**Conclusion:**

MZN staining procedure is reliable for the routine diagnosis of *Cryptosporidium*; cetyltrimethylammonium bromide extraction buffer and nested PCR targeting 18S rRNA gene are reliable and useful in epidemiological studies of this parasite.

## Introduction

The genus *Cryptosporidium* consists of protozoan parasites that invade the microvillus border of the gastrointestinal epithelium of all classes of vertebrates. The pathogenicity of *Cryptosporidium* varies with the species of parasite involved . Some species can infect many host species, some appear to be limited to particular animals groups, and others are known to infect one host species [1]. *Cryptosporidium* spp. infects a wide range of livestock animals and humans [2-8]. Those parasites are ubiquitous in the environment, and oocysts are extremely resistant to environmental conditions and many of the commonly used disinfectants [9]. Transmission can occur when material contaminated with viable oocysts is ingested [10]. The genus *Cryptosporidium* has specific morphological and biological features. However, morphological characterization within the genus is not always useful due to the lack of unique features among oocysts of the protozoan species. Host specificity can be very useful in supporting morphologic and genetic data when initially establishing the species taxonomy and the range of potential hosts [1].

*Cryptosporidium parvum* is recognized to infect several mammalian species as well as humans. Bovine sources of *C. parvum*, for example, have been reported to cause disease in laboratory personnel, veterinary students, and livestock workers [11-15]. Moreover, *Cryptosporidium* spp. infecting sheep and goats have been found to be of public health significance [16,17]. *Cryptosporidium hominis* (host specific) and *C. parvum* are the most commonly detected genotypes infecting humans [18,19]. *C. parvum* is of special importance as it can be of anthroponotic origin and zoonotic origin [20]. Thus, the reservoir hosts for *C. parvum* are domestic livestock and humans, while the reservoir host for *C. hominis* is human [21].

Polymerase chain reaction (PCR) and PCR-based techniques have gained arising significance in diagnosing bacterial and viral infections [22] and parasitic infections [23]. The availability of the genome sequence of *Cryptosporidium* spp. is a crucial step forward in the molecular identification of the species and genotypes of this parasite. Molecular characterization of *Cryptosporidium* species may be complicated. The DNA of the parasite is contained chiefly in the oocyst which possesses a vigorous wall difficult to break. Moreover, the feces contain large amounts of host DNA from sloughed intestinal cells, intestinal microflora, and other organisms [24,25]. Furthermore, some components of feces, such as heme, bilirubin, bile salts, and carbohydrates, weaken oocyst lysis, degrade DNA, and/or inhibit polymerase activity if co-extracted with the target pathogen DNA [26,27].

In Sudan, molecular data on *Cryptosporidium* species and genotypes in animals and humans are not well documented. Molecular studies can lead to better appreciation of the public and animal health importance of *Cryptosporidium* species and would enhance understanding of infection sources in humans in our country.

This study aimed to identify *Cryptosporidium* spp. infecting different animal species and humans and to compare between different isolates obtained. The study is an attempt to benefit from the availability of the sequence of this parasite in the activation and progress in research field on this organism and its pathogenicity.

## Materials and Methods

### Ethical approval

The present study was approved by the Institutional Ethics Committee (IEC), SUST/DSR/IEC/EA2/2014, Deanship of Scientific Research, Sudan University of Science and Technology (DSR-SUST).

### Specimen’s collection

Sixty-one microscopically *Cryptosporidium-* positive fecal specimens were obtained from cattle (n=6), sheep (n=2), and goats (n=48) in addition to five samples from immunocompromised patients. 30 negative fecal samples from goats were also included to be used as negative controls in PCR. All positive and negative samples were initially examined by a microscope using formol-ether concentration technique. Briefly, 1 g of feces was estimated using an applicator stick and then placed in a clean centrifuge tube containing 7 mL of 10% formalin. The sample was thoroughly broken up and mixed with an applicator stick. The steps were completed and followed by modified Ziehl–Neelsen (MZN) staining method according to OIE [10]. Infection intensity was quantitatively evaluated; positive and negative specimens were reported. A scoring system for positive samples (+≤5 oocysts per slide, ++=1-10 oocysts per field of view, and +++=11 or more oocysts per field of view) was used, based on the number of oocysts seen under the 100× objective lens. The oocysts per gram of feces were counted, morphological and morphometerical criteria were used, the size and shape index (length/width) of each oocyst was calculated, and then, the mean shape index of parasite was calculated [10]. All fecal specimens were stored at −20°C, without preservatives until used.

### Purification of oocysts from fecal samples

To obtain oocyst material and increase sensitivity of PCR reaction, *Cryptosporidium* oocysts were purified using sedimentation and floatation concentration techniques according to standard methods [10].

For sedimentation method, diethyl ether was added to Falcon tubes containing 2-3 g fecal specimen, mixed, and centrifuged at 1200× g for 5 min. The supernatant was discarded, and the pellets were then washed by distilled water, centrifuged 3 times, and then resuspended in phosphate-buffered saline [28].

For floatation concentration, oocyst suspensions from whole fecal specimens were prepared as described by Elwin *et al*. [29]. Briefly, oocysts were purified from the feces using saturated sodium chloride solution; 2-3 g of feces was added to 3 ml of flotation fluid in a 15 ml centrifuge tube (Falcon), mixed thoroughly, and then, sufficient flotation fluid was added to form a positive meniscus at the rim of the centrifuge tube, followed by centrifugation for 8 min at 1100× g [30]. The supernatant containing the oocysts was washed with distilled water, and the oocysts were resuspended in 1-2 ml of distilled water. Purified oocysts were then extracted through different DNA extraction methods.

### DNA extraction methods

#### DNA extraction using commercial kits

A commercial kit was used according to manufacturer’s instructions, with some modification to enhance cell rupturing. Briefly, 180-200 mg of each purified sample (n=30) was transferred into an Eppendorf tube and dissolved with 700 µL of ASL buffer of DNA Extraction Kit. Each sample was then exposed to seven cycles of freezing and thawing using liquid nitrogen (2 min) and boiling water (2-3 min) to disrupt oocysts cells. Afterward, the procedure was continued according to DNA Extraction Kit manufacturing instructions.

#### DNA extraction by cetyltrimethylammonium bromide (CTAB)

CTAB was used for the extraction of *Cryptosporidium* genomic DNA from 48 positive and 15 negative purified fecal samples. Briefly, the previously purified samples were transferred into 15 ml Falcon tubes containing 6 ml preheated (60°C) CTAB buffer. The solution was incubated in a water bath at 60°C for 30 min and inverted periodically. Followed by five cycles of freezing-thawing in liquid nitrogen and water bath (95°C) for 2-5 min each, then the tubes were left to cool at room temperature. 3 ml of chloroform isoamyl alcohol (24:1) was added to each tube, and the solutions were mixed gently and thoroughly for 10 min then centrifuged at 4000 rpm for 15 min. The aqueous phase was transferred by pipette into a new tube, and the precipitates were discarded. The addition of equal volumes of chloroform isoamyl alcohol (24:1) and the discarding of the precipitates were repeated twice. An equal volume of cold isopropanol was added to each tube and mixed gently then left to precipitate the DNA in a freezer for 30 min. The cool solutions were centrifuged at 4000 rpm for 15 min, then the supernatants were discarded, and equal volumes (3 ml) of 70% ethanol were added and then centrifuged at 4000 rpm for 5 min. The washing step by 70% ethanol was repeated twice. The supernatants were discarded, and the formed pellet was allowed to dry for 30 min at room temperature. The dried DNA pellets were resuspended in 30-50 μl of TE buffer (10 mM Tris and 1 mM EDTA [pH 8]) and stored at −20°C.

#### DNA extraction by phenol/chloroform

Forty positive and 15 negative *Cryptosporidium* fecal samples were extracted by this method. Briefly, purified fecal samples (pellet/suspension) were resuspended in 6 ml of TE (pH 8) in Falcon tubes. After vortexing for 5 min, samples were incubated at 75°C for 15 min, followed by five cycles of freezing-thawing in liquid nitrogen and water bath (65°C) for 2 min each. Then, DNA was extracted organically and also purified using conventional single step phenol/chloroform/isoamyl alcohol protocol as described by Abbaszadegan *et al*. [31]. After isopropanol precipitation, the colorless DNA pellet was collected and dissolved in 30-50 μl of TE buffer and incubated overnight at 4°C. The concentration of extracted DNA was measured by NanoDrop (Spectrophotometer, ND1000) and then stored at −20°C until used.

#### DNA amplification

PCR and nested-PCR were carried out as described by Kuzehkanan *et al*. [32] with few modifications, targeting *Cryptosporidium* 18S rRNA gene. Briefly, two pairs of specific primers for detecting all *Cryptosporidium* species were used to obtain PCR products of 347bp and 240bp in primary and secondary PCR, respectively.

For the primary PCR, Cry18S-S2, 5’ GGTGACTCATAATAACTTTACGG 3’ as forward and Cry18S-As2, 5’ ACGCTATTGGAGCTGG AATT AC 3’ as reverse were used, whereas nested PCR included Cry18S-S1, 5’ TAAACGGTAGGGTAT TGGCCT 3’ as forward and Cry18S-As1, 5’ CAGAC TTGCCCTCCAATTGATA 3’ as reverse. The PCR amplifications were performed with Maxime PCR PreMix Kit (i-Taq) in a 25 μl reaction mixture under standard conditions using G-STORM Thermal Cycler (England). The reaction mixture in primary PCR consisted of 20 μL distilled water, 3 μL of DNA template, and 1 μL of each primer (10 pmol/μL), while the secondary PCR amplification was performed using 1.5-2 μL of the primary amplification product, 1 μL of each primer (10 pmol/μL), and 21.5 μL of distilled water. The cycling conditions consisted of two steps: The first step - a primary PCR condition consisted of an initial denaturation at 94°C for 3 min followed by 14 cycles of denaturation at 94°C for 30 s, a touchdown annealing temperature from 60°C to 50°C for 30 s, and an extension for 1 min at 72°C each. The program was then continued for 35 cycles of 30 s at 94°C, 30 s at 55°C, 1 min at 72°C, and final extension for 5 min at 72°C, whereas the secondary PCR condition consisted of 94°Cfor 3 min, followed by 35 cycles comprising 1 min at 94°C, 1.30 min at 60°C, and 1 min at 72°C, then a final extension step of 10 min at 72°C.

Following amplification, secondary PCR products were subjected to electrophoresis on an ethidium bromide-stained 1.5% agarose gel. 100 base pair ladder was used to determine the length of each band. After approximately 30-45 min of electrophoresis, the gel was visualized by a documentation system (Gel Doc, Rain Low CCTVRMB192). Seventeen nested PCR products recognized to be *Cryptosporidium* positive were purified and sequenced in both directions in a commercial laboratory (Macrogen Scientific Services Co., Korea). Amplified sequences were contrasted with reference sequences using basic local alignment search tool.

### Phylogenetic analysis

Phylogenetic analyses were conducted in MEGA6 [33]. *Cryptosporidium* sequences were aligned by ClustalW alignment. All DNA sequences were analyzed by manual alignment editing and submission to the MEGA6 tree-building program. The evolutionary history was inferred using the neighbor-joining method [34]. The analysis involved 14 nucleotide sequences. All positions containing gaps and missing data were eliminated. *Theileria ovis* (KX273858.1) was used to root the constructed tree. The optimal tree was generated and bootstrap test (500 replicates) was applied. The confidence probability (multiplied by 100) showed that the interior branch length is >0, as estimated using the bootstrap test [35,36].

## Results

The detected oocysts (small spherical red bodies) were morphologically and morphometerically similar to *C. parvum*. The number of oocysts per gram of feces was <5 per smear in all goat, cattle, and sheep positive samples, whereas humans and calf fecal samples contained >5 oocysts per field (according to scoring system used). Although three methods of DNA extraction were used in the present study, only CTAB and phenol methods were successful in producing PCR products in subsequent PCR reaction. However, the lysis buffer - CTAB-extracted DNA - generated the sturdy and obvious band.

**Figure-1:**
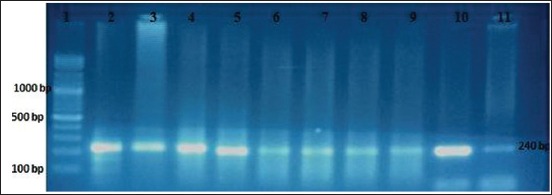
Representative ethidium bromide stained agarose gel showing polymerase chain reaction (PCR) products of *Cryptosporidium* DNA extracts from fecal samples. M: Molecular marker, Lanes: 2-11 nested PCR products.

Furthermore, the commercial kit expected to give best results showed negative results. Of 61 microscopic-positive fecal samples which passed DNA extraction, nested PCR amplified 56 (91.8%) samples which produced a 240 bp band (Figure-1). This indicated 100% specificity by microscopic method (MZN). In contrast, five positive samples (8.2%) detected by microscopic method (MZN) were found to be negative by nested PCR. Thus, nested PCR had 91.8% sensitivity in comparison with microscopic screening.

Thirty randomly selected negative samples that were tested with both primary and nested-PCR did not show any PCR-positive products. Concerning sequencing, of 17 sequenced PCR products, 14 (82.4%) samples passed the sequencing reaction, while the remnant 3 reactions (17.6%) were not successful. Alignment of the sequences obtained along with the reference sequences downloaded from GenBank suggested that all isolates from goats (n =8), sheep (n=1), cattle (n=2), and humans (n=3) belong to the *C. parvum* with 100% similarity to the reference sequence GQ259149.1 (Table-1).

**Table-1:** Selected *Cryptosporidium* spp. isolates matching current isolates.

Accession numbers	Identity (%)	Species	Source	Country
GQ259149.1	100	*Cryptosporidium parvum*	Hedgehog	Germany
AB513872.1	99	*Cryptosporidium parvum*	Calf	Egypt
KX668208.1	99	*Cryptosporidium parvum*	Elephant	India
KX151739.1	99	*Cryptosporidium parvum*	River water	Brazil
KX266232.1	99	*Cryptosporidium parvum*	Holstein	China
KU882702.1	99	*Cryptosporidium parvum*	Goat	Iraq
KU311869.1	99	*Cryptosporidium* spp.	Human	Lebanon
AJ493545.1	99	*Cryptosporidium parvum*	Human	Kenya
KU892564.1	99	*Cryptosporidium* spp. Horse	Human	Kenya

**Figure-2:**
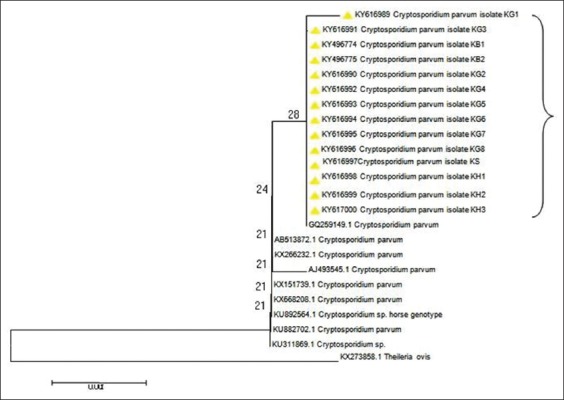
Phylogenetic relationships of the isolates examined in the current study (labeled) to different *Cryptosporidium* spp. as inferred by Neighbor-joining analysis of the 18s rRNA gene.

**Figure-3:**
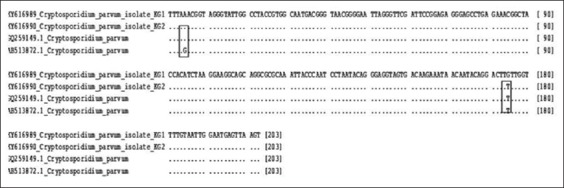
Alignment of *Cryptosporidium parvum* current isolates (KG1 and KG2) with selected *C. parvum* reference sequences showing different positions.

The phylogenetic analyses of the 18S RNA gene showed a close relatedness with the isolates examined in the current study and with some reference isolates previously identified (GQ259149.1) (Figure-2). Nevertheless, one isolate of goats (KY616989.1) differed from the other 13 isolates in a single position (Figure-3).

The 14 nucleotide sequences of the present study were deposited in GenBank under accession numbers: KY496774, KY496775, and KY616989-KY617000.

## Discussion

The present guide study was carried out to detect, identify, and contrast *Cryptosporidium* isolates from different animal species and humans. An MZN staining method was used to detect *Cryptosporidium* oocysts in fecal samples which were further confirmed by nested PCR. The study attempted Cryptosporidium DNA extraction using the commercial kit and two traditional methods. The commercial kits used failed to yield neither *Cryptosporidium* DNA nor positive PCR amplification. However, Hawash [37] used commercial kits for microscopically positive fecal samples containing larger numbers of oocysts and extracted *Cryptosporidium* DNA successfully. He also detected increased sensitivity and specificity from 60% to 100% after the introduction of freezing and thawing cycles and increasing temperature and duration of incubation. Thus, our negative findings might be due to the insufficient number of oocysts in fecal material and loss of DNA through subsequent column washes or precipitations, knowing that the kits are designed for small samples (180-200 mg).

On the other hand, all positive PCR products were produced from DNA samples extracted by traditional methods (CTAB and phenol/chloroform), indicating that the problem of low parasites number in samples was overcome by a large amount of fecal material used (up to 3 g) and preceded preparatory steps of freezing-thawing cycles enhancing probability of retrieving DNA with good quality and sufficient quantity. Nevertheless, CTAB-extracted DNA generated sturdy and obvious band when compared to phenol. Similarly, Zhao *et al*. [23] used four different methods to extract DNA from coccidian oocysts and reported that the CTAB-extracted DNA generated the strongest and clearest band.

Regarding the PCR amplification, in the present study, nested PCR advance was followed which is more reliable than single-step PCR as it prevents the possibility of receiving non-specific PCR bands and increases the chance of detecting low parasite concentrations. Our finding showed that nested PCR amplification consequences 91.8% (56/61) positive samples at 240bp. In contrast, Kuzehkanan *et al*. [32], using the same set of primers, reported that nested PCR was able to detect one more case which was negative by microscopic detection. Our finding may be again attributed to insufficient DNA or the presence of some inhibitors in primary PCR.

Concerning specificity and sensitivity, nested PCR as truthful PCR marker detected 91.8% (56/61) of microscopic positive samples, whereby five samples were suggested as false negative in nested PCR. In support of these findings, all samples negative by MZN were also negative through PCR. The current study argues that all samples missed by PCR were actually false negatives, and accordingly, our study disagreed with Morgan *et al*. [38] who considered samples detected by microscopy as false positive cases. In concordance to the current study, other studies [32,39] reported no false positive cases. In addition, Gawad *et al*. [40] reported that molecular diagnosis of human cryptosporidiosis is habitually superior to enzyme-linked immunoabsorbent assay (ELISA) and direct microscopy after concentration. Thus, our study strongly recommends preceding concentration techniques before specific staining (MZN) and DNA extraction to prevent false negative cases. Although PCR marker is the most sensitive method for the diagnosis of *Cryptosporidium*, concentration methods are considered more reliable in case of lacking PCR, especially in developing countries [41]. With regard to the inhibitory effect of coextracted material in fecal DNA samples, the preference of microscopy over PCR might be applicable to all DNA extracted from fecal samples.

In support of our findings in another data set, we evaluated MZN stain and antigen detection through ELISA in the diagnosis of *Cryptosporidium* in goats; we found that all positive samples by microscopic MZN were also positive by ELISA [42].

In the present work, nested PCR products were sequenced and aligned, and phylogenetic analysis was performed. Our results indicated that all examined isolates [14] are suggested to belong to the *C. parvum* with 100% similarity to the reference sequence (GQ259149.1) and 99% similarity to most of the *C. parvum* sequences previously deposited in GenBank. Accordingly, phylogenetic tree carried out showed a close relatedness among all isolates examined except one goat isolate (KY616989) which differed in a single position.

Our study suggests that *C. parvum* is the dominant *Cryptosporidium* spp. circulating in humans and animals in Khartoum state, Sudan, and considers these isolates to be adaptable for both zoonotic and anthroponotic transmissions, by direct or indirect contact. Our findings are supported by previous studies [10,18,19] reporting humans infected through both *C. parvum* and *C. hominis* and highlighting that humans exclusively infect humans and not naturally infect animals. In addition, Kosek *et al*. [21] reported that reservoir hosts for C. parvum are cattle, domestic livestock, and humans, while the reservoir host for *C. hominis* is human.

The use of PCR and animal models have provided the needed refined characterization of many former morphologically defined species. Likewise, in our study, all suspected *Cryptosporidium* isolates were confirmed by sequencing. In a separate study using the present human’s isolate and goats as animal model, we found that human’s isolate was pathogenic to goats causing different changes in hematological and biochemical profiles and even death (data not shown). These findings were further supported by the current partial 18S rRNA sequence analysis confirming and identifying the current *Cryptosporidium* isolates as *C. parvum*. Existing knowledge in Sudan indicates that molecular studies on *Cryptosporidium* spp. have been conducted neither in animals nor in humans. *C. parvum* was identified on the basis of morphology or serologically by demonstrating *C. parvum* antigen in feces using ELISA [8,43,44].

## Conclusion

This is the first report on molecular characterization of *Cryptosporidium* spp. in humans and animals in Sudan. The results have revealed a unique cryptosporidial infection in both humans and animals, where the *C. parvum* seems to be strongly adapted to both hosts. Animals seem to be the important reservoirs for the zoonotic *C. parvum*. Furthermore, the existence of the cryptosporidiosis in immunocompromised individuals as well as in selected animal species in the study area suggests a wide spread of infection in mammals including humans. Thus, further epidemiological studies are recommended to screen for infection rates and host variability of the *Cryptosporidium* spp. infection in Khartoum state.

## Authors’ Contributions

KYA: Collected samples, conducted experimental work, and drafted the manuscript; AAI: Developed research idea and contributed to conception and design; MAM: Contributed to molecular work, analysis, and manuscript writing; AAG: Supervised experimental work and approved the final manuscript. All authors read and approved the final manuscript except AAI. AAI died on January 24, 2015.
